# Novel Use of Endoscopic Hemospray to Achieve Hemostasis in Pulmonary Hemorrhage: A Case Series

**DOI:** 10.7759/cureus.53777

**Published:** 2024-02-07

**Authors:** Bharat S Bhandari, Aristides Jose Armas Villalba, Kathleen R Zavalla, David J Banay, George Eapen

**Affiliations:** 1 Department of Pulmonary Medicine, MD Anderson Cancer Center, Houston, USA

**Keywords:** pulmonary hemorrhage, massive hemoptysis, hemoptysis, hemostatic powder spray tc-325, fiberoptic flexible bronchoscopy, hemospray

## Abstract

This article presents two cases of pulmonary hemorrhage successfully managed using TC-325, a novel hemostatic powder commonly known as Hemospray. Originally approved for endoscopic hemostasis in gastrointestinal bleeding, Hemospray's application in endobronchial bleeding control has not been widely reported. The cases highlight its efficacy in achieving immediate and sustained hemostasis in peripheral pulmonary bleeding, where conventional bronchoscopic therapies may be ineffective. The absence of adverse effects and the rapid cessation of bleeding underscore the potential of Hemospray as a valuable tool in the bronchoscopist's arsenal, especially in life-threatening hemoptysis scenarios. The ease of application and quick hemostatic effects position Hemospray as a pragmatic solution for cases with challenging bleeding sources. While further studies are warranted to validate its efficacy and safety in a larger cohort, these cases advocate for considering Hemospray as a potential game-changer in the comprehensive management of hemoptysis, addressing limitations or risks associated with conventional interventions.

## Introduction

The Hemospray (Cook Medical) or TC-325 is a novel non-contact chemically inert, highly absorptive mineral agent hemostatic powder that was approved by the FDA for endoscopic hemostasis in May 2018. Since its introduction, Hemospray has been widely used for upper gastrointestinal bleeding with high rates of immediate hemostasis and low rebleeding percentages when compared to conventional endoscopic modalities [1]. Hemospray is an inorganic powder devoid of botanicals or animal proteins and functions through a dual mechanism. Upon contact with a bleeding site, it forms a prompt mechanical barrier, arresting bleeding by sealing the vessel wall. Simultaneously, its absorbent nature elevates local clotting factor concentration, promoting clot formation. Crucially, the powder's inability to be absorbed by mucosal tissues eliminates the risk of systemic toxicity, offering a safe and effective approach to managing mucosal bleeding. The use of Hemospray for endobronchial bleeding control has not been reported to our knowledge, likely because there has not been much awareness of the product in the interventional pulmonology community and the company’s reluctance to publicize an off-label use.

## Case presentation

**Figure 1:**
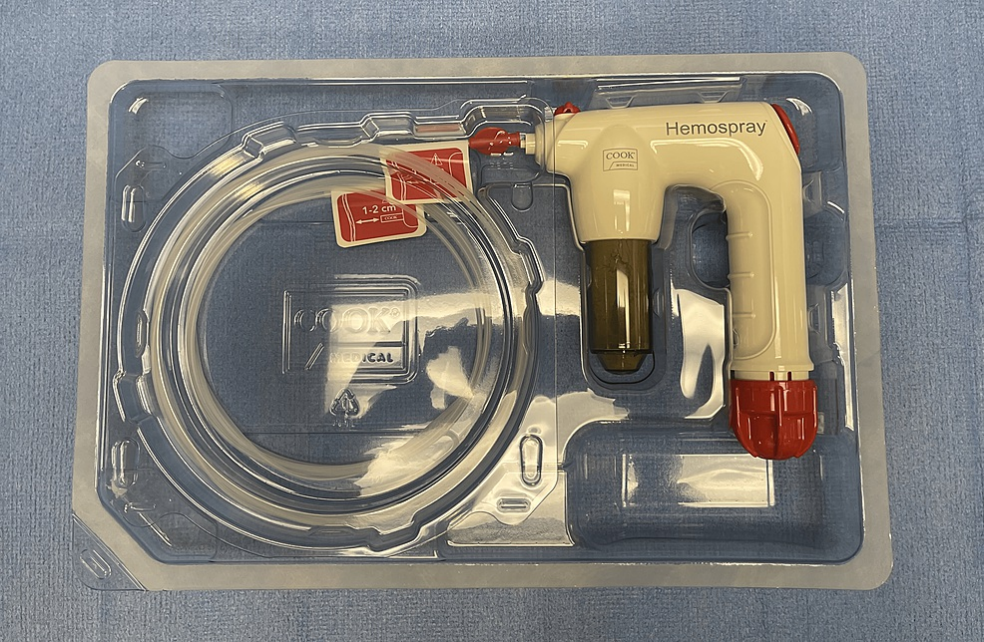
Hemospray (Cook Medical, North Carolina, USA)

We present the following cases of pulmonary hemorrhage that were controlled using the Hemospray (Figure 1).

Case 1

**Figure 2:**
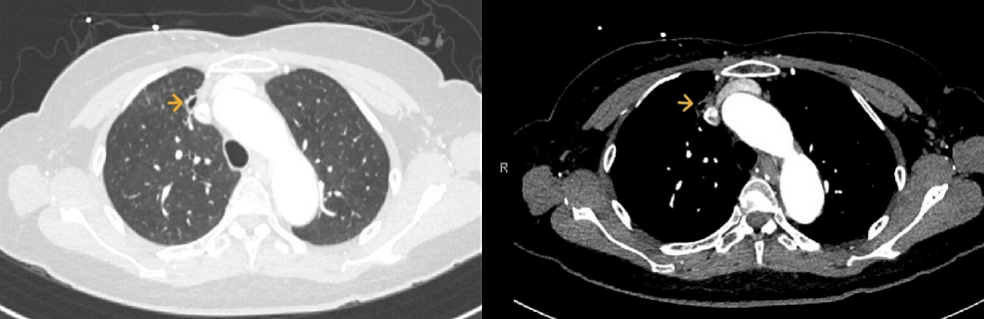
Chest CT Chest CT scan with contrast showed a small cavitary lesion in the right upper lobe and subtle bilateral lower lobe ground glass opacities.

**Figure 3:**
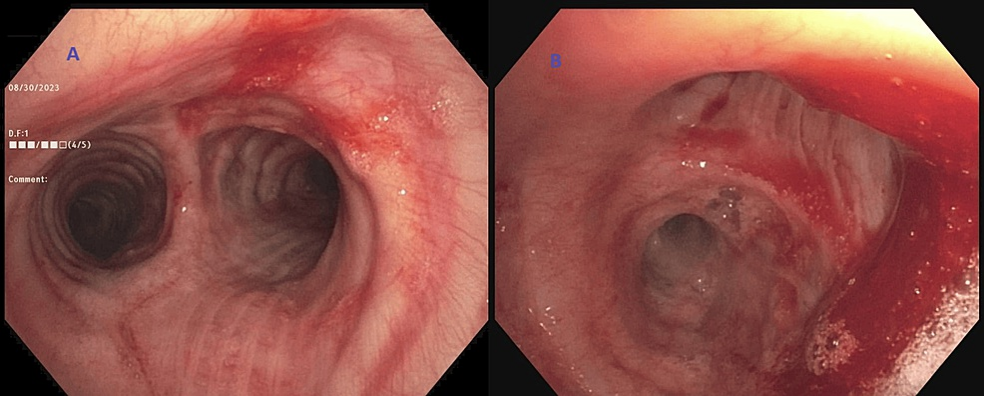
Flexible bronchoscopy (A) Showing blood in the tracheobronchial tree. (B) Blood clot in the right mainstem with oozing.

**Figure 4:**
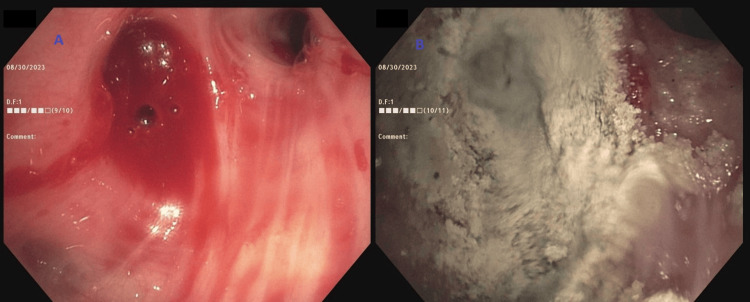
Flexible bronchoscopy intervention (A) Bleeding despite the instillation of iced saline and tranexamic acid. (B) Hemostasis was achieved following Hemospray instillation.

A 76-year-old lady recently diagnosed with stage IIB infiltrating ductal carcinoma of the left breast, who was undergoing neoadjuvant chemoimmunotherapy with pembrolizumab, paclitaxel, and carboplatin, presented to the hospital with two episodes of hemoptysis that she described as frank blood with small clots admixed with sputum. She had no prior pertinent pulmonary or cardiac history and was not on any anticoagulants. Initial chest CT scan with contrast showed a small cavitary lesion in the right upper lobe and subtle bilateral lower lobe ground glass opacities (Figure 2). A flexible bronchoscopy was performed to localize the bleeding source. Airway inspection revealed bloody secretions throughout the tracheobronchial tree, but no endobronchial disease was noted. Blood clots and active oozing was seen emanating from the right upper lobe anterior segment indicating peripheral pulmonary bleeding not amenable to direct bronchoscopic therapy (Figure 3). Initial attempts to control the oozing from the anterior segment of the right upper lobe with the instillation of iced saline and tranexamic acid proved ineffective and subsequently, Hemospray was instilled. The instillation resulted in immediate hemostasis (Figure 4). The patient tolerated the procedure well with complete resolution of hemoptysis and no recurrence occurred during the rest of her hospitalization.

Case 2

**Figure 5:**
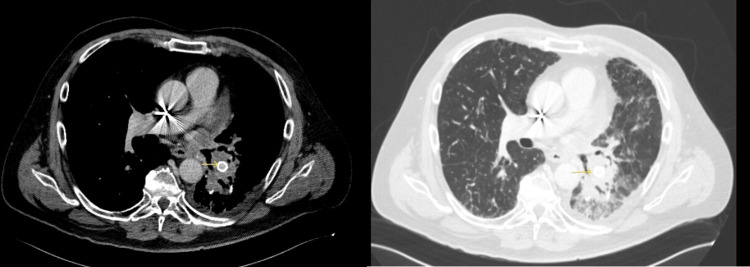
Chest CT Tumor involvement at the left secondary carina, proximity with the inferior pulmonary artery with the stent in place.

**Figure 6:**
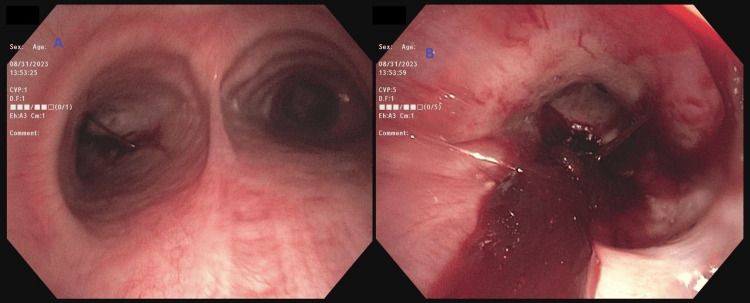
Flexible bronchoscopy (A) The main carina, blood seen coming from the left side. (B) Large blood clot occluding the left mainstem bronchus.

**Figure 7:**
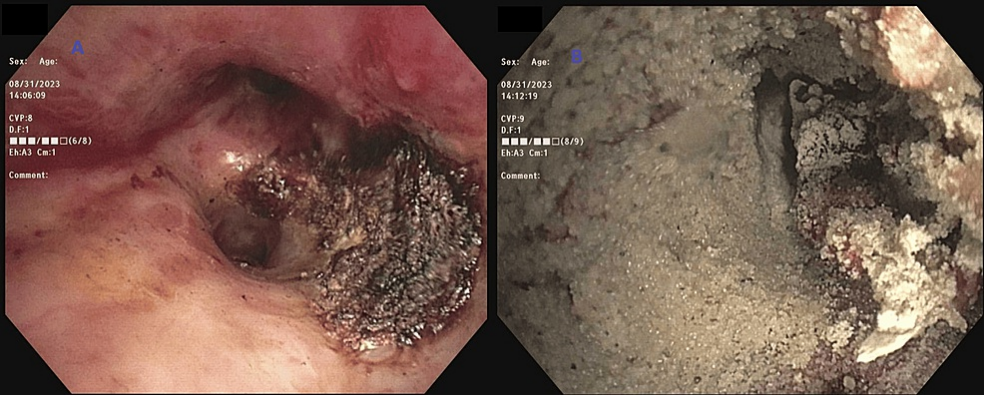
Flexible bronchoscopy intervention (A) Use of argon plasma coagulation with minor oozing. (B) Complete hemostasis was achieved using Hemospray.

A 62-year-old gentleman with metastatic carcinoid cancer of the lung previously treated with systemic therapy followed by radiation therapy was admitted with hemoptysis. The patient had multiple comorbidities including COPD on home oxygen, cardiomyopathy for which an AICD was placed, and a previous history of massive hemoptysis a year prior to the index admission that required bronchial artery embolization (BAE) and subsequent left inferior pulmonary artery stent placement. More recently he had required multiple admissions for hemoptysis and had undergone multiple bronchoscopies with argon plasma coagulation and laser therapy directed at bleeding areas noted in the left mainstem and left secondary carina. At the index admission, the patient reported multiple episodes of hemoptysis amounting to half a cup each time. Chest CT scan with contrast at admission showed tumor involvement at the left hilum, the left main bronchus extending along the left atrium around the inferior pulmonary artery and partially obstructing the superior pulmonary vein, with the previously placed inferior pulmonary artery stent in place (Figure 5). At bronchoscopy, a large blood clot almost completely occluding the left mainstem bronchus was seen and gently removed with suction (Figure 6). Subsequently, active bleeding was noted arising from the left upper lobe bronchus where diffuse nodular and extremely friable mucosa was seen infiltrating the entire left upper lobe bronchus. Argon plasma coagulation was used to fulgurate the accessible areas of the bleeding tumor, but this did not completely control the oozing. Hemospray was then used topically into the left upper lobe to obtain hemostasis and allow for fibrotic healing of the left upper lobe bronchus. This resulted in complete hemostasis (Figure 7). The patient tolerated the procedure well with complete resolution of hemoptysis and no recurrence occurred during the rest of his hospitalization.

## Discussion

Hemoptysis is a very distressing symptom for patients and a frequent cause for emergency room visits and hospitalization. Massive hemoptysis is a medical emergency and can be life-threatening. Definitions vary but commonly massive hemoptysis is defined as coughing up 100 to 600 cc of blood in a period of 24 h [2]. Some experts have posited that any amount of hemoptysis that impairs respiratory function should be treated with similar urgency [3]. In addition to the initial resuscitation measures, bronchoscopy is an integral part of evaluation and at times control of bleeding in a patient with hemoptysis, particularly when it is life-threatening. Bronchoscopy is particularly useful in cases where the hemoptysis originates from an endobronchial source that is visible to the bronchoscopist. Thermal therapies utilizing laser photocoagulation, electrocautery, and argon plasma electrocoagulation have all been described as highly effective in controlling hemoptysis in patients with bleeding endobronchial lesions. Temporary hemostasis has also been reported with cryotherapy or instillation of iced saline [4]. When an endobronchial source cannot be identified, the role of bronchoscopy becomes limited to localizing the source of bleeding for subsequent BAE or placing an endobronchial blocker to stabilize the patient while awaiting definitive therapy [5]. While BAE can be quite effective in certain cases, there is a small but definite risk of complications including spinal cord ischemia [6]. Several patients are also not candidates for BAE if the bleeding is intermittent and cannot be clearly localized at angiography. A topical agent that could promote rapid hemostasis in patients with peripheral sources for their hemoptysis would provide the bronchoscopist with a useful tool that we currently lack, resulting in improvement in patient care. TC-325, known as Hemospray, is a proprietary mineral-based hemostatic powder that, upon contact with blood, swiftly absorbs the liquid, initiating a clotting cascade and creating a barrier at the bleeding site [7]. It comes in a single-use device with a battery-powered pump that delivers the powder through a 2 mm diameter catheter. The catheter is inserted through the working channel of the bronchoscope and directed into the affected subsegment. Actuation for less than three seconds provides enough volume of powder to achieve hemostasis. There is extensive literature supporting the use of gastroenterologists for upper gastrointestinal bleeds [8]. There was an immediate cessation of bleeding in both of our cases. Our second case demonstrated the feasibility of combining endobronchial bleeding control using APC and peripheral bleeding control using Hemospray. Hemostasis was sustained over several days and with no need for additional interventions in both cases. Patients were extubated to room air with no post-procedure complications. 

## Conclusions

We described two cases with pulmonary hemorrhage where effective hemostasis was achieved using topical therapy: TC-325, known as Hemospray. In conclusion, the cases presented underscore the successful utilization of Hemospray, for the control of endobronchial bleeding, expanding its application beyond its established use in upper gastrointestinal bleeding. The absence of reported adverse effects in these cases, coupled with the immediate and sustained cessation of bleeding, highlights the potential of Hemospray as a valuable adjunct in the bronchoscopist's armamentarium for managing life-threatening hemoptysis. Traditional modalities such as thermal therapies have proven effective for visible endobronchial lesions, but Hemospray emerges as a promising option when dealing with peripheral pulmonary bleeding not amenable to direct bronchoscopic therapy. Its ease of application and rapid hemostatic effects offer a pragmatic solution for cases where the bleeding source is challenging to identify or access. In our restricted clinical encounters, Hemospray demonstrates optimal efficacy in managing distal bleeds that are not responsive to endoluminal therapy. Its clot-promoting characteristics make it particularly suitable for situations that would typically necessitate the use of an endoluminal balloon blocker. Hemospray generates a non-absorbable, gelatinous fibrinous material that, while effective in controlling bleeding, has the potential to cause luminal obstruction. Therefore, its preferential use is recommended in distal bleeds where the risk of life-threatening luminal obstruction is low, and patients can easily expectorate the formed clotted material. While acknowledging the need for further studies to validate its efficacy and long-term safety in a larger cohort, these cases advocate for the consideration of Hemospray as a potential game-changer in the comprehensive management of hemoptysis, especially in situations where conventional interventions may pose limitations or risks.
